# A New Anatomical Feature in Human Blood Corpuscles

**Published:** 1868-05-15

**Authors:** J. W. Freer

**Affiliations:** Prof. of Physiology in Rush Med. College


CHICAGO MEDICAL JOURNAL.

*Vol. XXV.—MAY* 15, 1868.—*No.* 10.

### DISCOVERY OF A NEW ANATOMICAL FEATURE IN HUMAN BLOOD CORPUSCLES.

BY J. W. FREER, M.D., PROF. OF PHYSIOLOGY IN RUSH MED. COLLEGE.

WITH the aid of Wale's Illuminator, by which blood corpuscles may be seen by reflected light, or as opaque objects, I have discovered that these bodies are not, as heretofore supposed, simply bi-concave discs; but, on the contrary, (by the aid of the above instrument) there may be seen a nipple-like eminence in the centre of the concavity of each well-formed disc. This papillary eminence is about $\frac{1}{10000}$ of an inch in diameter at the base. That it is a true anatomical form, and not a change incidental to desiccation, etc., is shown by its appearance at the instant of withdrawal of any given specimen, while the corpuscles are still plump, and smooth in all other respects.

By the use of the illuminator, I have also become convinced that among different individuals, there are always characteristic differences existing, concerning minute variations of feature, form, and perfection of the blood particles, and this obtains irrespective of apparent general physiological conditions.

The advantages offered by the illuminator for the satisfactory study of the external forms of histological elements is, I believe, as yet scarcely understood. This is probably due to the difficulties attending the acquirement of the successful use of the instrument. The method of its proper use once attained is rewarded by revelations at once unique and instructive. By its means all particles may be seen as solid objects, and as distinctively as pebbles on the sea shore. Blood corpuscles are as truly defined on the opaque slide as specimens of Boston crackers on a lacquered tea tray, and thus seen, are indeed objects of great beauty.

I do not at present propose to offer a theory concerning the signification of this heretofore undescribed anatomical feature of human blood discs; but one can scarcely avoid associating its presence with the long sought for nucleus of these bodies. The illuminator reveals the acknowledged nucleus of frog's blood cells as an eminence on their outer walls.

Over one year ago I called the attention of Dr. W. C. Hunt, of this city, to the peculiarity in human blood discs above related, but, notwithstanding, have delayed publication until, by repeated observations, the facts might be thoroughly confirmed or contradicted.

I take much pleasure in offering my obligations for Dr. W. C. Hunt's invaluable assistance in developing the proper method of using the illuminator.

